# Incidence of diabetes and its predictors in the Greater Beirut Area: a five-year longitudinal study

**DOI:** 10.1186/s13098-022-00833-w

**Published:** 2022-05-04

**Authors:** Mona P. Nasrallah, Martine Elbejjani, Lara Nasreddine, Hassan Chami, Hussein Ismaeel, Mohamad Fleifel, Fatima Al Zahraa Chokor, Hani Tamim

**Affiliations:** 1 Faculty of Medicine, Department of Internal Medicine, Division of Endocrinology, American University of Beirut Medical Center, Beirut, Lebanon; 2 Vascular Medicine Program, American University of Beirut, Beirut, Lebanon; 3 Faculty of Medicine, Clinical Research Institute, American University of Beirut Medical Center, Beirut, Lebanon; 4 Faculty of Medicine, Department of Internal Medicine, American University of Beirut Medical Center, Beirut, Lebanon; 5 Faculty of Agricultural and Food Sciences, Department of Nutrition and Food Sciences, American University of Beirut, Beirut, Lebanon; 6 Faculty of Medicine, Department of Internal Medicine, Division of Pulmonary and Critical Care, American University of Beirut Medical Center, Beirut, Lebanon; 7 Faculty of Medicine, Department of Internal Medicine, Division of Cardiology, American University of Beirut, Beirut, Lebanon; 8 College of Medicine, Alfaisal University, Riyadh, Kingdom of Saudi Arabia

**Keywords:** Type 2 Diabetes, Incidence, Lebanon, Beirut, MENA region, Predictors, Metabolic syndrome

## Abstract

**Background:**

Type 2 Diabetes (T2D) remains a world epidemic. Obtaining accurate estimates of its incidence and their predictors will aid in targeting preventive measures, allocating resources, and strategizing its management. The Middle East North Africa region has high T2D prevalence and rates of rise. Few incidence studies exist for the region, and none from Lebanon. The current study objective was to determine diabetes incidence and diabetes predictors in a community-based Lebanese sample. A secondary objective was to describe the metabolic control over time in adults with preexisting diabetes.

**Methods:**

This is a five-year (2014–2019) follow-up study on a random sample of 501 residents of the Greater Beirut area. Out of 478 people eligible to participate in the follow-up study, 198 returned (response rate 39.5%). Assessment included medical history, anthropometric measures, food frequency, sleep, and lifestyle questionnaires. Laboratory data included glycemic indices (fasting glucose and HbA1C) and other biological markers. The diagnosis of probable diabetes (PD) was based on one abnormal test for either fasting glucose ≥ 126 mg/dL or HbA1C ≥ 6.5% or having history of diabetes.

**Results:**

The incidence of diabetes was 17.2 (95% CI 9.6–28.7) per 1000 person-years. Cardiometabolic risk factors independently associated with diabetes were: older age, higher BMI, family history of diabetes, metabolic syndrome, higher CRP and triglyceride level; whereas an independent predictor of diabetes was previous BMI.

In addition, the 42 participants with preexisting diabetes had worsening of their metabolic profile over a five-year period.

**Conclusions:**

The incidence of diabetes was high as compared to some reported world rates, and in line with the high prevalence in the MENA region. The risk was highest in those with positive family history and the presence of the metabolic syndrome or its components. Preventive measures should particularly target participants with that specific risk profile. This becomes particularly important when observing that metabolic control gets worse over time in individuals with diabetes.

**Supplementary Information:**

The online version contains supplementary material available at 10.1186/s13098-022-00833-w.

## Introduction

Type 2 diabetes (T2D) is a world epidemic, with increasing prevalence reported since the latter half of the twentieth century [1]. Thirty-year projected prevalence rates were largely surpassed in less than half the time predicted. As an example, in the year 2000, Hossein et al. projected the total number of people with diabetes to reach 366 million by 2030 [1]. However, the world prevalence of diabetes was estimated at 371 million by 2012 and reached 463 million in 2019 [2].

Diabetes prevalence provides real time data, which is essential in strategizing diabetes management, as well as comparing it across different areas of the globe. Worldwide, the age- standardized prevalence is 8.3%, being highest in the Middle East North Africa (MENA) region at 12.2% and lowest in Europe at 6.3% [3]. In Lebanon, a small country of the MENA region, we have reported a prevalence in 2014 of 15.0% in community-dwelling adults, which stood on the high side for that region (slightly lower than the Gulf countries, but higher than North Africa) [4]. Our reported prevalence was higher than prior reports conducted in Lebanon five and ten years earlier [5, 6]. However, the increasing prevalence rates can be due to an aging population, as well as better diabetes survival, rather than a true higher incidence of diabetes mellitus [7].

Diabetes incidence provides a more accurate estimate about the behavior of the condition and its risk factors; and over time, incidence trends would refine projections about future diabetes rates. A recent systematic review assessing diabetes trends showed that in some areas of the world, there is stability and even regression in diabetes incidence [7]. The review mostly included high-income countries, which is where the data was available from.

However, looking at prevalence studies, the greatest rates of rise in diabetes involve the low to middle-income countries such as in the Indian continent, South East Asia, and MENA regions [2]. Incidence rates for these regions are either not available or highly variable. As an example, in Kerala (India), the incidence of diabetes in adults was 24 per 1000 person-years (2007–2017) and in Zhejiang (China) 2.7 per 1000 person-years for the same 10-year follow-up period [8, 9]. In Tehran (Iran), the incidence over 2001–2009 time period was 10.6 per 1000 person-years [10] and in Turkey in a nationwide study (1998–2005) it was 11.0 and 12.4 for women and men, respectively per 1000 person-years [11]. In comparison, rates were less variable and ranged from 6.9 globally in the US [12] to 8.7 in South-West Germany [13] and 10.8 in the province of Asturias (Spain in 1999–2005) [14], per 1000 person-years.

On one hand, the demography in the low- to middle-income populations shares some similarity with rapid population aging [15], increased urbanization, and nutrition transition. On the other hand, more health expenditure is spent on tertiary rather than primary diabetes care, influencing diabetes rates and survival [2].

Given the magnitude of the epidemic and factors influencing it in the MENA region, it becomes important to assess the true rise in diabetes mellitus by measuring its incidence and associated risk factors.

As a follow-up on our previous cross-sectional study reporting on the prevalence of diabetes in Beirut, the capital of Lebanon [4], we report on the development of diabetes at the 5-year interval and its predictors in the same cohort of participants. Additionally, we report on the follow-up metabolic status in those who had diabetes at baseline.

## Materials and methods

This study is based on the Greater Beirut Area Cardiovascular Cohort study, which was originally initiated in 2014. The details of the study have been published elsewhere [4, 16–18]. Briefly, it was a cross-sectional, community-based study using multistage probability sampling, of 501 adult Lebanese men and women residing in Beirut. The study recruitment was conducted over a 3-month period from March until May 2014. To be eligible to participate in the study, subjects had to be older than 18 years old, Lebanese, and residing in the Greater Beirut area. Subjects were excluded from participating in the study if they were dialysis patients, pregnant women, subjects with intellectual inability to understand the study description and procedure and to provide informed consent, and/or those working in a plastic or other chemical company. The study was approved by the Institutional Review Board of the American University of Beirut. Participants provided informed consent and agreed to be part of follow-up studies. Data collected included demographic, socioeconomic, lifestyle-related, medical history, anthropometric measures and laboratory tests.

*Follow-up study* The follow-up study was conducted 5 years after the baseline study (February to May 2019). The follow-up study was approved by the Institutional Review Board at the American University of Beirut, and written informed consent was obtained again from the participants.

### Study procedures

All participants who had agreed at baseline to be called again were contacted by phone. They were offered to return for a follow-up visit. In case, they were unable or unwilling to, a brief health questionnaire was conducted over the phone, enquiring about the development of major non-communicable conditions. For those willing to participate, a follow-up visit was scheduled in the same premises as the initial study, at the Department of Nutrition and Food Sciences at the American University of Beirut. Participants were instructed to come after a 10-h fast, and to bring their current medications with them. All questionnaires and measures were conducted following the study’s standardized protocol and in a confidential manner, by trained Collaborative Institutional Training Initiative (CITI) certified staff. Only participants who came to the follow-up study were considered responders and included in the final analysis.

### Data collection

Participants underwent face-to-face interview, anthropometric assessment, and laboratory studies. Most of the information collected was similar to those collected in 2014:

- (1) Demographic and socioeconomic: age; gender; area of residence; marital status (categorized as married, single, or other being separated/divorced/widowed); education (divided into no schooling/primary school, intermediate, secondary/technical, or university); occupation; crowding index (defined as the average number of people per room, excluding kitchen and bathroom [19]; and income bracket per family (categorized as (USD per month): less than <600, 600-999.9, or more than 1000 USD. For analysis purposes, the first two brackets were grouped into one).
- (2) Lifestyle habits: dietary intake (using a validated 80-item culture-specific semi-quantitative Food Frequency Questionnaire (FFQ) [20]; physical activity (using the short version of the International Physical Activity Questionnaire (IPAQ) [21]; smoking (with current defined as any daily smoking, regardless of the number of cigarettes, narghileh, or e-cigarettes); alcohol intake (defined as any intake); and caffeine intake. Sleep was assessed using the Berlin questionnaire for obstructive sleep apnea [22] and the Epworth Sleepiness scale (ESS). Regarding the FFQ, IPAQ, Berlin, and ESS scales, we have used at baseline and upon follow-up the same validated Arabic version of these scales [23–26].
  Specific nutrition items known to be associated with T2D were selected from the detailed questionnaire, for their association either as negative predictors (caffeine [27], dairy [28, 29], fiber partially estimated by the surrogate measure of fresh fruit and vegetable intake [30] or as positive predictors (glycemic index and load [31], sugar-sweetened beverages and juices [32]).
- (3) Medical history: family history, review of systems, medication intake, and all chronic illnesses (diabetes mellitus, cardiovascular disease, hypertension, dyslipidemia, cancer, thyroid disease, etc.). Specific diabetes-related questions included whether the participant had diabetes or not. If the answer was yes, then enquiry about the duration and management was recorded.
- (4) Anthropometric measures: weight and height (using a calibrated scale); waist circumference (using a standardized method [33]); body composition (using bioimpedance analyzer (Inbody Body Composition Analyzer Inbody 230; sitting heart rate and blood pressure using a digital sphygmomanometer)). Blood pressure was obtained twice, and the average was recorded to the nearest millimeter of mercury. Body mass index (BMI) was calculated and categorized according to the World Health Organization classification for overweight and obesity [33].
- (5) Laboratory measures: fasting plasma glucose (FPG), hemoglobin A1C (HbA1c), serum creatinine, lipid profile, C-reactive protein (CRP), insulin, thyroid stimulating hormone (TSH), 25-hydroxyvitamin D, and urinary microalbumin to creatinine ratio (ACR). Insulin resistance was calculated using the homeostasis model of assessment of insulin resistance (HOMA-IR) [34].

The metabolic syndrome was defined using the harmonized criteria of the International Diabetes Federation (IDF) [35]. Participants were classified as having the Metabolic syndrome if they met three of the following five risk factors: elevated triglyceride level (≥ 150 mg/dL), low HDL level (< 40 mg/dL for men and < 50 mg/dL for women), elevated blood pressure (systolic ≥ 130 and/or diastolic ≥ 85 mm Hg), elevated FPG level (≥ 100 mg/dL), and elevated waist circumference (≥ 94 cm for men and ≥ 80 cm for women).

### Laboratory procedures and assays

Blood (15 mL) and urine were obtained. A total of 5 ml of whole blood were split into two purple top tubes, one frozen for future studies, and the other refrigerated and sent on the same day for HbA1c measurement. The remainder of the blood (10 ml) was centrifuged and the serum was split into several 1 mL Eppendorf tubes. One tube was refrigerated and sent on the same day for glucose measurement. The remaining tubes were kept frozen at  − 80 °C for future assays. A fingerstick glucose was obtained at the same time as the laboratory tests.

Laboratory tests were subsequently done using the following methodology: HbA1c by HPLC (Bio-Rad); FPG by Enzymatic method (Cobas 6000, Roche); Serum and Urine Creatinine by the Jaffe rate method (Cobas 6000, Roche); Urine microalbumin by Immunoturbidimetry (Cobas 6000, Roche); TSH, 25(OH) and vitamin D by electrochemiluminescence Immunoassay (ECLIA) (Cobas e 411, Roche), and insulin by immunoassay (Cobas e 411, Roche). Levels of triglycerides, HDL-C, and total cholesterol were measured using enzymatic colorimetric method (Cobas 6000, Roche), and of LDL-C using Coupled Classic precipitation (Cobas 6000, Roche). CRP was measured by Immunoturbidimetric assay (Cobas 6000, Roche).

### Glycemic definitions

The same glycemic definition and cut-offs were used for the current study as that of the baseline study, and according to the American Diabetes Association (ADA) guidelines [36].

Participants were classified into one of the following categories as follows:

- Probable diabetes (PD), if they had history of diabetes (answered “yes” to the question: Do you have diabetes?), are on T2D medications, and/or have either FPG ≥ 126 mg/dl, or HbA1c ≥ 6.5% (48 mmol/mol)
- High risk of diabetes (HR), if they had no history of diabetes (answered “no” to the question: Do you have diabetes?), were not on T2D medications, and have FPG between 100 and 125 mg/dL and/or HbA1c between 5.8 and 6.49% (40 and 47 mmol/mol)
- Low risk of diabetes (LR), if they had no history of diabetes (answered “no” to the question: Do you have diabetes?), were not on T2D medications, and have FPG < 100 mg/dl and HbA1c < 5.8% (40 mmol/mol)

Because the ADA requires two abnormal tests for the diagnosis, we created a category labelled ‘definite diabetes’ DD which is included within the PD but is more stringent, requiring both tests to be abnormal and/or the answer ’yes’ to diabetes.

Additionally, because the phenotype and progression of dysglycemia fit well with phenotype of Type 2 Diabetes (T2D), the term PD and T2D are used interchangeably in the remainder of the paper.

### Statistical analysis

Data were entered into the Statistical Package for Social Sciences (SPSS, version 22) which was used for data cleaning, management, and analyses. Characteristics of participants were described using number and percent for categorical variables and mean and SD ( ±) for continuous and normally distributed variables, and median (IQR) for continuous not normally distributed variables. Comparison between different groups was carried out using chi-square, t-tests, and ANOVAs, as applicable. Moreover, appropriate non-parametric tests were used when sample size was small, mainly Fisher’s exact test, and Mann–Whitney test.

Incidence rates of diabetes were calculated by dividing the number of new cases by the total person-time between baseline and study-visit for those who were diabetes free at baseline (more specifically those who were ND or RD at baseline). Incidence rates were computed for probable diabetes and for being at-risk for diabetes; age-stratified incidence rates for all these incidence rates were also computed.

We then compared subjects with incident diabetes to those who were diabetes free at follow-up with regards to their baseline characteristics and their follow-up characteristics. We used stepwise multivariable regression analyses to obtain adjusted predictors for diabetes incidence. The models included factors that were either identified by statistical significance or clinical significance. P-value of 0.1 was set for the entry of potential predictors into the model, whereas a P-value of 0.2 was set for removal from the model. Results were presented as odds ratio (OR) and 95% confidence interval (CI). We use the term ‘predictors’ when referring to factors associated with diabetes from the 2014 data; and we used the term ‘cardiometabolic risk factors’ for factors associated with diabetes from the 2019 data (as we could not infer the temporal link in the latter).

Moreover, assessment of changes in metabolic control between baseline and follow-up, for those who had diabetes at baseline, was carried out using paired sample t-test.

P-value < 0.05 was used to indicate statistical significance.

## Results

**Fig. 1:**
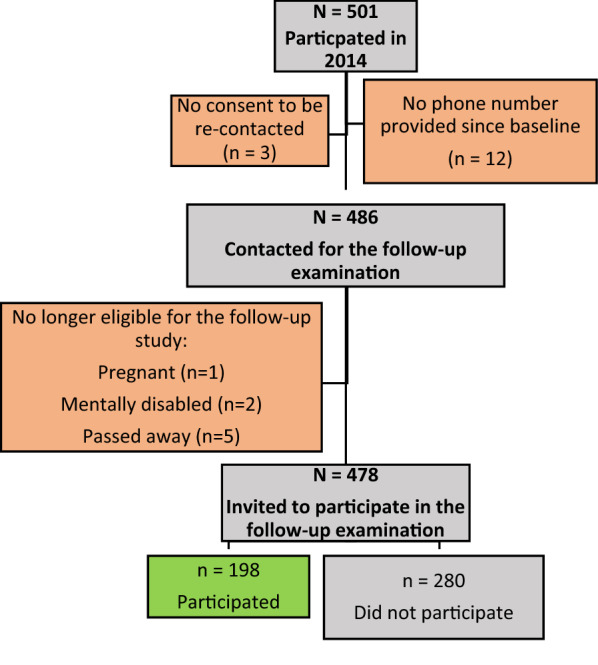
Response Rate Flow Diagram

Out of 501 participants, 486 consented to be re-contacted and had a phone number available. Out of the 486, 8 subjects were no longer eligible to be part of the study. The remaining sample eligible for this follow up study was 478, out of which 198 agreed to take part in this study. Overall, the net participation rate after 5 years was 39.5%; n = 198 (participants) as compared to 303 (non-participants). Primary reasons for non-participation, in addition to the previously cited reasons, were wrong phone number (36.1%), no answer (17.9%), being too busy (17.5%) not interested (15.7%), too ill (8.9%) or having moved/traveled (3.9%). Figure 1 presents the flow diagram of participants’ inclusion in this study.

### Differences between participants and non-participants

Participants and non-participants were compared with respect to their baseline characteristics (Table 1). Participants were older (46.97 ± 13.3 versus 44.3 ± 16.0 years, p-value = 0.046) and had higher BMI (30.0 ± 5.8 versus 28.5 ± 5.7 kg/m^2^, p-value = 0.005). They were more likely to be married, and to have a lower socioeconomic status. However, they did not differ with respect to gender, smoking status, and physical activity. They had a smaller proportion of alcohol consumers (14.1 versus 22.1%, p-value = 0.03). They did not differ in the prevalence of diabetes (15.2 versus 11.2%, p-value = 0.2), or dyslipidemia, and tended to have lower hypertension prevalence than non-responders.

**Table 1:** Comparison of baseline characteristics at baseline in 2014 between subjects who responded to the follow-up visit in 2019, compared to those who did not

Variables		Responders N = 198	Non-responders N = 303	p-value
Age	Mean (± SD)	47.0 ± 13.3	44.3 ± 16.0	0.046
Gender	Female	127 (64.1)	195 (64.4)	0.96
BMI	Mean (± SD)	30.0 ± 5.8	28.5 ± 5.7	0.005
Marital status	Married	148 (74.7)	184 (60.7)	< 0.0001
	Single	21 (10.6)	77 (25.4)	
	Other	29 (14.6)	42 (13.9)	
Income (USD per month)	< 600	68 (34.7)	85 (28.2)	0.002
	600–999.9	79 (40.3)	91 (30.2)	
	≥ 1000	38 (19.4)	92 (30.6)	
	I don’t know/no answer	11 (5.6)	33 (11.0)	
Education	No schooling/primary school	77 (39.1)	104 (34.6)	0.08
	Intermediate school	61 (31.0)	75 (24.9)	
	Secondary school/technical diploma	44 (22.3)	83 (27.6)	
	University degree	15 (7.6)	39 (13.0)	
Current cigarette smoker	Yes	78 (39.4)	138 (45.5)	0.17
Current narghileh smoker	Yes	56 (28.3)	86 (28.4)	0.98
Current alcohol drinker	Yes	28 (14.1)	67 (22.1)	0.03
Coffee drinker	Yes	159 (80.3)	244 (80.5)	0.95
Physical activity	None	29 (14.6)	50 (16.5)	0.58
	Any	169 (85.4)	253 (83.5)	
Probable diabetes or PD	Yes	30 (15.2)	34 (11.2)	0.20
systolic blood pressure mmHg	Mean (± SD)	122.0 ± 19.0	121.2 ± 19.4	0.64
diastolic blood pressure mmHg	Mean (± SD)	75.7 ± 10.2	74.1 ± 9.7	0.07
Hypertension diagnosis	Yes	38 (19.2)	81 (26.7)	0.052
Hypertension treatment	Yes	36 (18.2)	75 (24.8)	0.08
Dyslipidemia diagnosis	Yes	51 (25.8)	69 (22.8)	0.44
Dyslipidemia treatment	Yes	41 (20.7)	53 (17.5)	0.37

Significance was considered at a p value < 0.05

### Diabetes incidence

Among the participants, 42 had probable diabetes in 2014, and were therefore excluded from further analysis pertaining to incidence. Moreover, five patients did not have glycemic data available in 2019 and thus, were excluded from the analyses. The diabetes analysis was conducted on 151 participants, who took part of this study and did not carry a diagnosis of diabetes at baseline. Out of these, 13 subjects developed new PD, with eight who self-reported having diabetes on the medical history questionnaire, and five who were diagnosed using the laboratory cut-offs. Therefore, proportion of unknown PD was 38%.

Diabetes incidence was 17.2 (95% CI 9.6–28.7) per 1000 person-years (Table 2). The incidence was markedly higher in the subgroup who were HR in 2014 which was 29.3 per 1000 person-years, as compared to 5.3 per 1000 person-years among those who were LR in 2014 (Table 2). When this incidence was stratified by the current age of participants (in 2019), the incidence was higher among those who were ≥ 40 years.

**Table 2:** The incidence of diabetes among responders at follow-up in 2019, per 1000 person-years, among two groups (LR and HR) *

	Total		Among those with No Diabetes in 2014 Low risk (LR)		Among those at Risk for Diabetes in 2014 High risk (HR)	
	Incidence rate/1000* person-year (95%CI)		Incidence rate/1000* person-year (95%CI)		Incidence rate/1000* person-year (95%CI)	
	n	N = 151	n	N = 76	n	N = 75
No diabetes	63	83.4 (64.7 – 106.1)	47	123.7 (91.9 – 163.1)	16	42.7 (25.3 – 67.8)
Probable diabetes	13	17.22 (9.6 – 28.7)	2	5.26 (0.88 – 17.39)	11	29.3 (15.4 – 50.9)
Prediabetes	75	99.3 (78.7 – 123.8)	27	71.05 (47.8 – 101.9)	48	128.0 (95.4 – 168.3)
Age < 40 years		N = 39		N = 25		N = 14
No diabetes	24	123.1 (80.7 – 180.3)	19	152.0 (94.2 – 233.0)	5	71.4 (26.2 – 158.3)
Probable diabetes	1	5.1 (0.2 – 25.3)	1	8.0 (0.4 – 39.5)	0	0
At Risk diabetes	14	71.8 (40.9 – 117.6)	5	40.0 (14.7 – 88.7)	9	128.6 (62.7 – 235.9)
Age ≥ 40 years		N = 112		N = 51		N = 61
No diabetes	39	69.6 (50.2 – 94.3)	28	109.8 (74.4 – 156.6)	11	36.1 (19.0 – 62.7)
Probable diabetes	12	21.4 (11.6 – 36.4)	1	3.9 (0.2 – 19.3)	11	36.1 (19.0 – 62.7)
Prediabetes	61	108.9 (84.0 – 139.0)	22	86.3 (55.4 – 128.5)	39	127.9 (92.2 – 173.0)

^*^ LR: Low risk of Diabetes or euglycemic; HR High risk for diabetes or prediabetes

It is noteworthy to mention that among those who were at HR in 2014 (n = 27), a larger proportion (59%) regressed to no diabetes (or euglycemia), than those who progressed (41%) to PD (Additional file 1: Table S1). Factors that were related to progression versus regression were older age, higher obesity indices, higher CRP, higher HbA1C, and HOMA-IR. They were also physically less active, consumed more fruits, but did not differ in other nutritional parameters (Additional file 1: Table S1).

### Risk factors for the development of diabetes

Table 3 compares the risk factors at baseline in 2014, and the subsequent development of diabetes at the 2019 follow-up visit. No gender difference was noted. Those who developed diabetes were older (50.7 ± 10.8 versus 44.3 ± 13.3 years), but the difference did not reach statistical significance (p-value 0.12). However, they were more obese as reflected by BMI (33.5 ± 4.2 versus 28.6 ± 5.1 kg/m^2^, p-value = 0.001), as well as higher percent body fat, and waist circumference. Subjects who developed diabetes at follow-up tended to have higher income and fared better in terms of education (both non-significant). There were less smokers among the group with diabetes (38.5 versus 67.4%, respectively) and more former smokers (38.5 versus 10.1%, respectively). As would be expected, those who developed diabetes had higher glycemic indices at baseline (HbA1C, fasting plasma glucose), but not in the diabetic range. They also had more insulin resistance by HOMA-IR (8.9 (4.9) versus 6.3 (3.5), p-value = 0.002), higher inflammatory marker CRP (13.0 (16.0) versus 9.0 (7.0) mg/L, p-value = 0.03), higher triglyceride (183.0 (81.0) versus 106.5 (96.0) mg/dL, p-value = 0.03), and lower HDL-C. Similarly, the metabolic syndrome, was a significant predictor of diabetes with twice as many having the syndrome among the diabetes group (84.6 versus 44.9%, p-value = 0.008).

**Table 3:** Comparison of probable diabetes vs low risk (LR)/high risk (HR) of diabetes with all the variables at baseline in 2014

Variables		Low/high risk of diabetes n = 138	Probable diabetes N = 13	p-value
Demographic				
Age	Mean (± SD)	44.3 ± 13.3	50.7 ± 10.8	0.12
Gender	Female	86 (62.3)	7 (53.8)	0.56
Marital status	Married	108 (78.3)	10 (76.9)	0.10
	Single	19 (13.8)	0 (0.0)	
	Other	11 (8.0)	3 (23.1)	
Income	< 1000 $	104 (80.6)	9 (69.2)	0.30
	≥ 1000 $	25 (19.4)	4 (30.8)	
Education	No schooling/primary school	51 (37.2)	2 (15.4)	0.37
	Intermediate school	41 (29.9)	6 (46.2)	
	Secondary school/technical diploma	33 (24.1)	3 (23.1)	
	University degree	12 (8.8)	2 (15.4)	
Lifestyle habits				
Smoking	Never	31 (22.5)	3 (23.1)	0.01
	Current	93 (67.4)	5 (38.5)	
	Former	14 (10.1)	5 (38.5)	
Current alcohol drinker	Yes	23 (16.7)	1 (7.7)	0.69
Coffee drinker	Yes	113 (81.9)	10 (76.9)	0.71
Sleep apnea index	Low risk	87 (71.9)	3 (33.3)	0.02
	High risk	34 (28.1)	6 (66.7)	
Any physical activity		120 (87.0)	10 (76.9)	0.32
Physical activity status	Low	61 (44.2)	7 (53.8)	0.69
	Moderate	49 (35.5)	5 (38.5)	
	High	28 (20.3)	1 (7.7)	
Specific nutrition items consumption per 24 h				
Coffee consumption/ mgrams	Median (IQR)	225.9 (283.5)	161.2 (243.3)	0.55
Coffee cups/ number	Median (IQR)	2.7 (3.6)	3.0 (3.4)	0.92
Dairy consumption / grams	Median (IQR)	119.6 (181.0)	209.8 (251.5)	0.53
Fruits/grams	Median (IQR)	197.4 (190.3)	203.6 (204.2)	0.75
Vegetables/ grams	Median (IQR)	110.8 (177.1)	164.1 (209.3)	0.78
Fruits/KCal	Median (IQR)	125.1 (131.7)	139.9 (124.0)	0.86
Vegetables/ KCal	Median (IQR)	77.8 (63.3)	59.2 (64.6)	0.84
Glycemic load	Median (IQR)	208.9 (123.2)	186.2 (272.5)	0.87
Glycemic index	Mean (± SD)	61.5 ± 7.2	64.1 ± 9.0	0.22
Sweetened beverages/ grams	Median (IQR)	127.4 (227.8)	188.6 (291.6)	0.92
Fruit juices fresh /grams	Median (IQR)	37.6 (78.4)	11.6 (33.4)	< 0.0001
Medical history				
Hypertension diagnosis	Yes	12 (8.7)	2 (15.4)	0.35
Hypertension treatment	Yes	11 (8.0)	1 (7.7)	1.00
Dyslipidemia diagnosis	Yes	23 (16.7)	4 (30.8)	0.25
Dyslipidemia treatment	Yes	18 (13.0)	3 (23.1)	0.40
Obesity indicators and vital signs				
Systolic blood pressure mmHg	Mean (± SD)	118.8 ± 17.2	121.3 ± 18.3	0.66
Diastolic blood pressure mmHg	Mean (± SD)	74.4 ± 10.3	77.3 ± 9.7	0.40
Heart rate (bpm)	Mean (± SD)	77.7 ± 10.0	76.3 ± 11.6	0.46
BMI	Mean (± SD)	28.6 ± 5.1	33.5 ± 4.2	0.001
Waist circumference (cm)	Mean (± SD)	93.2 ± 12.3	108.2 ± 10.3	< 0.0001
Body fat (kg)	Mean (± SD)	27.5 ± 10.4	37.2 ± 7.6	0.001
Muscle Mass (kg)	Mean (± SD)	26.7 ± 6.4	29.7 ± 7.9	0.20
Metabolic syndrome	Yes	62 (44.9)	11 (84.6)	0.008
Laboratory measures				
HBA1C (%)	Mean (± SD)	5.5 ± 0.4	5.8 ± 0.2	0.001
Fasting plasma glucose (mg/dL)	Mean (± SD)	98.3 ± 9.0	104.5 ± 9.3	0.03
Fingerstick glucose (mg/dL)	Mean (± SD)	100.2 ± 8.1	105.5 ± 8.1	0.04
HOMA-IR	Median (IQR)	6.3 (3.5)	8.9 (4.9)	0.002
Creatinine (mg/dL)	Mean (± SD)	0.8 ± 0.2	0.8 ± 0.1	0.52
Insulin (IU/mL)	Median (IQR)	25.6 (12.6)	36.5 (14.7)	0.004
C-reactive protein (mg/dL)	Median (IQR)	9.0 (7.0)	13.0 (16.0)	0.03
HDL-C (mg/dL)	Median (IQR)	49.0 (19)	42.0 (12.0)	0.01
LDL-C (mg/dL)	Median (IQR)	109.5 (42.0)	104.0 (35.0)	0.63
Triglyceride (mg/dL)	Median (IQR)	106.5 (96.0)	183.0 (81.0)	0.03
Vitamin D (ng/dL)	Mean (± SD)	16.0 ± 8.6	16.5 ± 8.6	0.52
Urine microalbumin/creatinine ratio (ug/gm)	Median (IQR)	58.6 (133.8)	84.4 (55.1)	0.85

Significance was considered at a p value < 0.05

^*^ND no Diabetes or euglycemic, *RD* at risk for diabetes or prediabetes

The association with these variables was consistently replicated when doing the same comparison (diabetes and low risk/high risk of diabetes) using the follow-up visit in 2019 (Table 4). More specifically, we found the following associated cardiometabolic risk factors: age, obesity indices, and smoking. Per definition, the glycemic indices were in the frank diabetic range for the diabetes group, and the metabolic status was overall consistently worse than the nondiabetic group with respect to CRP (12.0 (15.0) versus 7.0 (5.0)mg/L, p-value = 0.003), triglycerides (164.0 (78.0) versus 119.0 (69.0)mg/dL, p-value = 0.01), HDL-cholesterol (42.1 ± 10.1 versus 50.0 ± 13.5 mg/dL, p-value = 0.04) and HOMA-IR (6.0 (4.1) versus 2.4 (2.5), p-value = 0.02). Consistent with these cardiometabolic risk factors, the metabolic syndrome remained significant (Table 4).

**Table 4:** Comparison of probable diabetes vs low risk (LR)/high risk (HR) of diabetes with all the variables at follow-up in 2019

Variables		Low/high risk of diabetes n = 138	Probable diabetes N = 13	p-value
Demographic				
Age	Mean (± SD)	49.3 ± 13.3	55.7 ± 10.8	0.12
Gender	Female	86 (62.3)	7 (53.8)	0.56
Marital status	Married	109 (79.6)	11 (84.6)	0.64
	Single	11 (8.0)	0 (0.0)	
	Other	17 (12.4)	2 (15.4)	
Income	< 1000$	90 (65.7)	10 (76.9)	0.54
	≥ 1000 $	47 (34.3)	3 (23.1)	
Education	No schooling/primary school	37 (26.8)	3 (23.1)	0.95
	Intermediate school	45 (32.6)	4 (30.8)	
	Secondary school/technical diploma	42 (30.4)	5 (38.5)	
	University degree	14 (10.1)	1 (7.7)	
LIfestyle habits				
Smoking	Never	31 (22.5)	3 (23.1)	0.03
	Current	97 (70.3)	5 (38.5)	
	Former	10 (7.2)	5 (38.5)	
Current Alcohol Drinker	Yes	18 (48.6)	2 (40.0)	1.00
Coffee Drinker	Yes	118 (85.5)	10 (76.9)	0.42
Sleep apnea index	Low risk	87 (63.0)	7 (53.8)	0.56
	High risk	51 (37.0)	6 (46.2)	
Any Physical activity		109 (79.0)	7 (53.8)	0.08
Physical activity status	Low	70 (50.7)	8 (61.5)	0.78
	Moderate	49 (35.5)	4 (30.8)	
	High	19 (13.8)	1 (7.7)	
Specific nutrition items consumption per 24 h				
Coffee consumption/ mgrams	Medin (IQR)	225.1 (251.8)	209.3 (454.5)	0.37
Coffee cups/ number	Median (IQR)	2.1 (4.1)	2.7 (2.2)	0.81
Dairy consumption / grams	Median (IQR)	121.9 (158.2)	169.5 (191.8)	0.30
Fruits/grams	Median (IQR)	300.5 (286.8)	427.0 (210.3)	0.20
Vegetables/ grams	Median (IQR)	216.1 (174.0)	147.9 (2.05)	0.70
Fruits/Kcal	Median (IQR)	203.1 (206.0)	234.5 (172.9)	0.76
Vegetables/ Kcal	Median (IQR)	77.4 (62.9)	43.7 (72.0)	0.83
Glycemic load	Median (IQR)	203.8 (128.3)	75.6 (103.6)	0.86
Glycemic index	Mean (± SD)	60.7 ± 6.6	61.9 ± 8.3	0.54
Sweetened beverages/ grams	Median (IQR)	96.3 (177.9)	78.6 (223.5)	0.67
Fruit Juices Fresh /grams	Median (IQR)	37.7 (82.0)	32.2 (66.1)	0.99
Medical history				
Family history of diabetes		64 (46.4)	11 (84.6)	0.009
HTN diagnosis	Yes	37 (26.8)	3 (23.1)	1.00
HTN treatment	Yes	28 (20.3)	6 (46.2)	0.07
Dyslipidemia diagnosis	Yes	44 (31.9)	5 (38.5)	0.76
Dyslipidemia treatment	Yes	36 (26.1)	6 (46.2)	0.19
Obesity indicators and vital signs				
Systolic blood pressure mmHg	Median (IQR)	117.0 (21.3)	121.0 (26.8)	0.53
Diastolic blood pressure mmHg	Mean (± SD)	77.4 ± 9.8	79.8 ± 12.7	0.64
Heart rate (bpm)	Medan (IQR)	74.0 (16.0)	73.0 (23.0)	0.45
BMI	Median (IQR)	29.3 (6.9)	33.2 (6.9)	0.01
Waist circumference (cm)	Mean (± SD)	97.2 ± 13.4	111.0 ± 10.8	0.01
Body fat (kg)	Mean (± SD)	28.1 ± 10.7	38.2 ± 10.3	0.002
Muscle Mass (kg)	Mean (± SD)	27.6 ± 6.5	30.2 ± 7.6	0.23
Metabolic syndrome	Yes	58 (42.0)	11 (84.6)	0.006
Laboratory measures				
HBA1C (%)	Median (IQR)	5.4 (0.0)	6.3 (1.0)	< 0.0001
fasting plasma glucose (mg/dL)	Median (IQR)	99.5 (15.0)	133.0 (27.0)	< 0.0001
Fingerstick glucose (mg/dL)	Median (IQR)	97.5 (14.0)	121.0 (26.5)	< 0.0001
HOMA-IR	Median (IQR)	2.4 (2.5)	6.0 (4.1)	0.02
Creatinine (mg/dL)	Mean (± SD)	0.8 ± 0.2	0.80 ± 0.2	0.84
Insulin (IU/mL)	Median (IQR)	9.6 (8.0)	16.9 (7.0)	0.001
C-reactive protein (mg/dL)	Median (IQR)	7.0 (5.0)	12.0 (15.0)	0.003
HDL-C (mg/dL)	Mean (± SD)	50.0 ± 13.5	42.1 ± 10.1	0.04
LDL-C (mg/dL)	Median (IQR)	105.5 (41.0)	104.0 (48.0)	0.84
Triglyceride (mg/dL)	Median (IQR)	119.0 (69.0)	164.0 (78.0)	0.01
Vitamin D (ng/dL)	Median (IQR)	11.2 (11.0)	13.0 (19.0)	0.33
Urine microalbumin/creatinine ratio (ug/gm)	Median (IQR)	46.9 (65.0)	30.8 (64.8)	0.23

Significance was considered at a p value < 0.05

The dietary factors known to be associated with diabetes (coffee, dairy, fiber, glycemic index/load, and sugar-sweetened beverages) did not differ between diabetic and non-diabetic participants at both baseline and follow-up (Tables 3 and 4). However, fresh fruit juice (categorized as a separate item from sweetened beverages) was higher among the non-diabetic versus the diabetic group at baseline in 2014 (37.6 (78.4) versus 11.6 (33.4) grams per day, p-value < 0.0001), respectively (Table 3). This difference was no longer observed at follow-up in 2019, with the probable diabetes group increasing their fresh fruit juice intake (32.2 (66.1) versus 37.7 (82.0) grams per day, p-value = 0.99) (Table 4). In parallel to the increase in fresh fruit juice intake among the diabetic participants, there was a corresponding decrease in the consumption of sweetened beverages between 2014 (188.6 (291.6) grams per day) and 2019 (78.6 (223.5) grams per day).

With respect to lifestyle, the group with diabetes was consistently more sedentary at baseline and follow-up, even though this difference did not reach statistical significance (Tables 3 and 4). They also had higher risk of sleep apnea as compared to the group without diabetes (66.7 versus 28.1%, respectively, p-value = 0.02) at baseline (Table 3). The trend, even though no longer significant remained at follow-up (Table 4).

Subjects who developed diabetes were twice as likely to have a first degree relative with T2D at follow-up in 2019 (84.6% versus 46.4%, p-value = 0.009, for diabetic versus nondiabetic participants, respectively) (Table 4).

### Predictors of diabetes and associated cardiometabolic risk factors

To assess the predictors of development of diabetes, multivariate analyses included the following variables: age; gender (reference: male); BMI; Systolic Blood Pressure, C-reactive protein; Triglyceride; and sleep apnea (reference: low). Independent predictors for the development of diabetes using the baseline data in 2014 were BMI and the Metabolic syndrome. However, due to small sample size, the Metabolic syndrome risk estimate was not precise (OR = 6.41, 95% CI 1.242, 33.082) and was therefore not included in the model. As far as BMI, for each increase of 1 kg/m^2^, the OR for developing diabetes over the next 5 years was 1.20 (95% CI 1.07 – 1.34).When using the 2019 data, family history was strongly associated with incidence of diabetes, but due to the small sample size, the estimate for Family history was not precise either (OR = 15.22, 95% CI 1.74, 133.05) so it was removed along with Metabolic syndrome and family income from the list of variables included in the model. Finally, cardiometabolic risk factors that were associated with incident diabetes were age, BMI, CRP, and triglycerides. The independent predictors for 2014 and independent cardiometabolic risk factors for 2019 are presented in Table 5.

**Table 5:** Stepwise logistic regression of predictors (at baseline 2014) and associated cardiometabolic risk factors (at follow-up 2019) of probable diabetes

	Probable diabetes		
		OR (95%CI)	P-value
2014 (baseline)	BMI	1.20 (1.07–1.34)	0.002
2019 (follow-up)	Age	1.06 (1.00–1.12)	0.06
	BMI	1.16 (1.02–1.32)	0.02
	C-reactive protein	1.13 (1.02–1.25)	0.02
	Triglyceride	1.01 (1.00–1.02)	0.01

Variables included in the model were: Age; gender (reference: male); BMI; Systolic Blood Pressure, C-reactive protein; Triglyceride; and sleep apnea (reference: low)

Significance was considered at a p value < 0.05

### Progression of diabetes among the 2014 cohort

To evaluate the change in metabolic control over time for established cases, the metabolic indices were compared between the two visits for the 42 participants with preexisting diabetes who returned for follow-up (Table 6). The overall duration of diabetes at follow-up was a median of 120.0 months (range 84.0 –179.0). The average HbA1C and fasting glucoses increased slightly despite near doubling of those taking oral hypoglycemic therapy. Moreover, the proportion of participants with poorly controlled diabetes (HbA1C ≥ 9%) nearly doubled from 11.9 to 19.0%. The most commonly used hypoglycemic therapy was metformin alone or in combination with sulfonylurea. The average BMI remained in the obese range. The blood pressure and lipid indices remained stable, however with an increase in anti-hypertensive (69% versus 40.5%) and statin intake (40.5% versus 14.3%) for 2019 and 2014, respectively. Additionally, there was progression of microalbuminuria (Table 6).

**Table 6:** For the group with Probable Diabetes in 2014, comparison of characteristic variables between baseline in 2014 and follow-up in 2019

	2014 N = 42	2019 N = 42	p-value
Age (years)	54.5 ± 11.0	59.0 ± 10.9	< 0.0001
Lifestyle and anthropometric			
Smoking			1.00
Never	9 (21.5)	9 (21.5)	
Current	25 (59.5)	25 (59.5)	
Former	8 (19.0)	8 (19.0)	
Sleep apnea index			1.00
Low risk	17 (50.0)	19 (45.2)	
High risk	17 (50.0)	23 (54.8)	
Any Physical activity	36 (85.7)	31 (73.8)	0.18
Physical activity status			0.86
Low	21 (50.0)	23 (54.8)	
Moderate	17 (40.5)	16 (38.1)	
High	4 (9.5)	3 (7.1)	
BMI	33.3 ± 7.1	32.8 ± 7.0	0.14
Systolic blood pressure mmHg	131.6 ± 21.7	134.7 ± 22.5	0.47
Diastolic blood pressure mmHg	79.2 ± 10.0	79.6 ± 11.0	0.87
Medication intake			
Anti-hypertensive intake	17 (40.5)	29 (69.0)	0.002
Cholesterol medication	6 (14.3)	17 (40.5)	0.003
Diabetes medications			
None (diet only) Oral hypoglycemic#	25 (59.5) 16 (38.1)	11 (26.2) 29 (69.0)	0.001
Insulin	0 (0.0)	0 (0.0)	−
Combination *	1 (2.4)	2 (4.8)	1.00
Laboratory measures			
HBA1C (%)	7.4 ± 1.7	7.5 ± 1.9	0.56
Proportion HbA1C			0.29
< 7	21 (50.0)	20 (47.6)	
7–9	16 (38.1)	14 (33.3)	
≥ 9	5 (11.9)	8 (19.0)	
fasting plasma glucose (mg/DL)	147.4 ± 44.3	165.0 ± 59.5	0.04
Triglyceride (mg/dL)	178.8 ± 66.2	185.4 ± 72.8	0.42
LDL (mg/dL)	109.4 ± 39.3	105.4 ± 42.2	0.53
HDL-C (mg/dL)	46.0 ± 13.1	45.0 ± 12.3	0.50
Urine microalbumin/creatinine ratio (ug/gm)	18.1 (32.8)	31.7 (56.8)	0.05

Significance was considered at a p value < 0.05

^*^Combination refers to oral hypoglycemic agents with insulin

^#^Oral hypoglycemic classes were for the majority sulfonylureas with metformin [8], or with pioglitazone [1], metformin alone [6], or single DPP4 inhibitor [1] in 2014. In 2019, the most common classes remain metformin [8] with sulfonylureas [8]; however, there is an increase in use of DPP4 inhibitors in combination with other classes [7], pioglitazone [1], new use of SGLT2 inhibitors [2] and others (3)

## Discussion

During a five-year follow-up of a community cohort with 501 adult participants, out of which retention was nearly 40%, the incidence of diabetes was 17.2 per 1000 person-years. This constitutes the first diabetes incidence study for Lebanon, and enriches data for the MENA region, which are largely limited. The incidence obtained stands higher than other community-based incidence studies available from the MENA region, namely Iran (10.6) and Turkey (11.7), as well as with high income Mediterranean countries such as Spain (10.8) per 1000 person-years [10, 11, 14]. However, it stands closer to the incidence obtained from the United Arab Emirates of 16.3 per 1000 person-years [37]. The average BMI among these 362 adult men and women in the Emirati sample was 31.7 kg/m2, which is close to the BMI of our study population. There are limited incidence data from other countries in the MENA region, However, by extrapolating to prevalence, the higher incidence obtained is in parallel with the higher prevalence of 15.0% we have previously obtained [4] and which is closer to that of the Arab Gulf countries [3]. The higher rate is concerning and may be linked to a more morbid population. Therefore, it is important to conduct a nationwide incidence study.

It is equally important to determine the risk factor for incident diabetes. Predictors for the development of diabetes were in line with the traditional risk factors, the most important ones being obesity and the presence of prediabetes or being at high risk [14]. However, the presence of prediabetes alone was not enough to predict the progression into diabetes, as the majority regressed to normoglycemia if they were younger, leaner, and more physically active. It is when coupled with obesity and metabolic derangements that progression, rather than regression took place. It is therefore important to provide aggressive lifestyle change in individuals with prediabetes, obesity, metabolic syndrome, high triglyceride, low HDL-C, and high CRP. In other words, these are the individuals with metabolically ‘unhealthy’ obesity [38]. There is data to support that metabolic derangements occur on a spectrum, and that individuals who have developed such a profile have ‘ectopic’ fat which is associated with insulin resistance and the subsequent development of the metabolic syndrome [39].

Having first degree relatives with T2D doubles the risk for diabetes, over and above the metabolic derangements and should present one additional identifier to intervene with diabetes prevention measures [40].

Looking at the risk factor change between baseline and follow-up; it is interesting to note that there is a high level of consistency. This observation is reassuring firstly because it supports the validity of our data, and secondly because it provides confidence that a single capture of risk factors in cross-sectional studies likely reflect relationships with these risk factors across time. The minimal change in associated risk factors (such as smoking, obesity, low level of physical activity, and sleep disturbance) also reflects the difficulty in adopting a healthy lifestyle and improving anthropometrics. Yet, the evidence is clear that such a change is the basis for metabolic improvement. Intervention studies in such risk profile population have been well-documented, for example in the Diabetes Prevention Study [41] and have been adapted to an Arab American population in the United States [42]. Such an intervention study is highly needed in Lebanon, to assess feasibility of implementation and outcome in our population. Even though genetics, culture, and nutrition habits may share similarities between our local population and Arab-Americans, there are factors which remain peculiar to Lebanon (specifically Beirut), such as the poor neighborhood walkability [43], stressful living conditions, high noise level [44], high level of environmental pollution, and poor air quality, to mention a few. After the completion of the current study, there has additionally been the catastrophic August 4, Beirut Port explosion in a year already burdened by COVID-19 adding to all the above stressors [45].

Certain risk factors did not follow their traditionally established association with incident diabetes. For example, smoking tended to be negatively associated with incident diabetes unlike the currently established understanding [46, 47]. However, the relationship between smoking and diabetes is complex as was shown in a multiethnic study conducted on 6000 adult participants [47]. In the latter study, former smokers had a higher association than current smokers. This was also the case in our study, likely representing a behavioral change in this higher vascular risk group or potential survival bias with the smokers studied being different than general smoking population. Overall, the rates of ever smoking were very high in our population at 65% when compared to other studies [47] which may additionally decrease the discriminating ability to identify a potential link with incident diabetes.

Similarly, coffee intake did not have a protective association as described in the literature [48]. There is evidence that coffee is rich in antioxidants and caffeine stimulates insulin release. However, epidemiologic studies describing the benefit have not been conducted in the MENA region where culturally coffee is consumed in an unfiltered, heavily brewed and concentrated fashion, known as ‘Turkish’ coffee. The latter preparation has been associated with an adverse lipid profile [49] as compared to filtered ‘American’ coffee. No such distinction has been made with respect to glycemic profile. However, the different preparation procedure does result in lower antioxidant content [50], and therefore may lose its glycemic benefit. Again, the rates of coffee drinkers were high at 80% in our study, affecting the ability to detect an effect. There is a need for more dissection of the relationship between coffee consumption and effect on diabetes because of a heterogeneity of factors [48].

No other dietary risk factors emerged, but one interesting observation was the increased consumption of fruit juice among the group with diabetes, with an equal reduction in the consumption of sweetened beverages between baseline and follow-up. It is possible that the participants who developed diabetes over the course of five years have made dietary adjustments which they perceived as ‘healthy’, in relation to their metabolic derangements [51].

One important finding in our study was the evolution of diabetes among those already diagnosed with PD in 2014. We have observed that there is little improvement and rather worsening of the metabolic profile in terms of glycemic control, body weight, and albuminuria. The blood pressure and lipids were on target, however at the expense of near doubling the rate of medication intake. The latter finding is in line with previous findings from Lebanon where the direction is towards worsening control and more complications once diabetes is established [52]. Furthermore, there is a high degree of underutilization of insulin, a finding corroborated by previous studies we conducted [4, 53].

Of similar concern as the lack of improvement of diabetes control is the high proportion of undiagnosed diabetes (38%), in line with the numbers described for the MENA region [54]. From a preventive perspective, our study found a high incidence of diabetes, with more than a third undiagnosed, and worsening control of those already diagnosed. Our findings call for a change at the system level which will result in reduction of risk factors, more use of medication where appropriate, especially insulin, and earlier detection of the condition [55].

### Strengths and limitations

Among the strengths of our study is being prospective and following the same rigorous data collection methods. On the other hand, our study is limited in the relatively low rate of responders. The response rate of 39.5% five years later, in Lebanon, a country characterized by a high level of population mobility, coupled with the standardized methodology, are not major threats to the validity of the study [56]. Indeed, most of the no-response was due to inability to reach participants based on their past contact information. In addition, the differences between responders and nonresponders were small in magnitude and did not include a systematic difference (e.g., responders were slightly older, had higher BMI, but lower hypertension). Nonetheless, the possibility of a selection bias must be acknowledged; the older age and higher BMI could have resulted in a more selective sample with a higher observed diabetes incidence, as well as its relation to these predictors. We note however, that the proportion of participants with unknown diabetes was similar to our previously reported study and to the MENA region (44%), further suggesting that participants who have returned are not specifically different from the baseline sample. There is however limited generalizability of our findings to the national level as the study was restricted to Beirut, and the incidence obtained was not adjusted to the population demographics. Moreover, we have made the assumption that participants who developed diabetes had T2D based on their profile and risk factors. However, the possibility of other types such as T1D, LADA, or MODY cannot be ruled out. Finally, even though our findings are in line with the literature in terms of increasing incidence and its predictors, the small sample size makes the results in the present study more exploratory in nature, and ideally should constitute a steppingstone to a larger, more representative study.

## Conclusions

In summary, diabetes incidence in a community-based cohort of Lebanese participants was elevated, considered similar to the higher endemic countries from the MENA region, and in line with the higher prevalence observed in Lebanon. In addition to the numbers obtained, given the projections that the fastest rate of rise of diabetes is in the MENA region, the trend in the incidence rate would be crucial to assess whether our incidence is similarly expanding. The only diabetes predictor in our study was high BMI, whereas associated cardiometabolic risk factors were components of the metabolic syndrome.,. Prevention should target these risk factors aggressively, particularly in individuals with familial predisposition; especially that the metabolic path for those with already established diabetes is a cause for concern.

## Supplementary Information

**Additional file 1:** Comparison between participants with RD at baseline in 2014 who either regressed to ND or progressed to PD at follow-up in 2019.

## Data Availability

The datasets used and/or analyzed during the current study are available from the corresponding author on reasonable request.

## Abbreviations

ACR: Urinary microalbumin to creatinine ratio
ADA: American Diabetes Association
BMI: Body mass index
CRP: C-reactive protein
FFQ: Food Frequency Questionnaire
FPG: Fasting plasma glucose
HbA1C: Hemoglobin A1C
HDL: High density lipoprotein
HR: High risk of diabetes
HOMA-IR: Homeostasis model of assessment of insulin resistance
IPAQ: International Physical Activity Questionnaire
LDL: Low density lipoprotein
LR: Low risk of diabetes
MENA: Middle East North Africa
PD: Probable Diabetes
SPSS: Statistical Package for Social Sciences
T2D: Type 2 Diabetes
TSH: Thyroid stimulating hormone
