# Supplementary material for: Incidence of diabetes and its predictors in the Greater Beirut Area: a five-year longitudinal study
Source: Diabetol Metab Syndr. 2022 May 4;14:67. doi: 10.1186/s13098-022-00833-w (PMC9066987; doi:10.1186/s13098-022-00833-w)
Supplement: Supplementary file 1 — Additional file 1: Comparison between participants with RD at baseline in 2014 who either regressed to ND or progressed to PD at follow-up in 2019. [file 13098_2022_833_MOESM1_ESM.docx]

| **2014 data** |  | **HR in 2014 who regressed to LR in 2019, n=16** | **HR in 2014 who progressed to PD in 2019,n=11** | **p-value** |
| --- | --- | --- | --- | --- |
| **DEMOGRAPHIC** | | | | |
| **Age (years)** | **Mean (±SD)** | 42.0 ± 15.1 | 51.8 ± 10.6 | 0.07 |
| **Gender** | **Female** | 11 (68.8) | 5 (45.5) | 0.26 |
| **Marital status** | **Married** | 10 (62.5) | 8 (72.7) | 0.16 |
|  | **Single** | 4 (25.0) | 0 (0.0) |  |
|  | **Other** | 2 (12.5) | 3 (27.3) |  |
| **Income (USD per month)** | **<1000**  **≥1000** | 12 (80.0) | 8 (72.7) | 1.00 |
|  |  | 3 (20.0) | 3 (27.3) |  |
| **Education** | **No schooling/primary school**  **Intermediate school**  **Secondary school/technical diploma**  **University degree** | 5 (31.3) | 2 (18.2) | 0.51 |
|  |  | 8 (50.0) | 4 (36.4) |  |
|  |  | 2 (12.5) | 3 (27.3) |  |
|  |  | 1 (6.3) | 2 (18.2) |  |
| **LIFESTYLE HABITS** | | | | |
| **Smoking** | **Never** | 3 (18.8) | 2 (18.2) | 0.01 |
|  | **Current** | 13 (81.3) | 4 (36.4) |  |
|  | **Former** | 0 (0.0) | 5 (45.5) |  |
| **Current Alcohol Drinker** | **Yes** | 4 (25.0) | 1 (9.1) | 0.30 |
| **Coffee Drinker** | **Yes** | 13 (81.3) | 9 (81.8) | 1.00 |
| **Sleep apnea index** | **Low risk** | 9 (69.2) | 2 (28.6) | 0.16 |
|  | **High risk** | 4 (30.8) | 5 (71.4) |  |
| **Any Physical activity** |  | 14 (87.5) | 8 (72.7) | 0.37 |
| **Physical activity status** | **Low** | 4 (25.0) | 7 (63.6) | 0.08 |
|  | **Moderate** | 9 (56.3) | 4 (36.4) |  |
|  | **High** | 3 (18.8) | 0 (0.0) |  |
| **Specific nutrition items** | | | | |
| **Coffee consumption/ mgrams** | **Mean (±SD)** | 247.6 ± 215.3 | 270.2 ± 278.2 | 0.98 |
| **Coffee cups/ number** | **Mean (±SD)** | 3.1 ± 2.9 | 3.9 ± 4.4 | 0.72 |
| **Dairy consumption / grams** | **Mean (±SD)** | 141.2 ± 140.3 | 200.5 ± 130.4 | 0.16 |
| **Fruits/grams** | **Mean (±SD)** | 134.1 ± 80.8 | 269.7 ± 175.8 | 0.009 |
| **Vegetables/ grams** | **Mean (±SD)** | 201.2 ± 112.3 | 247.4 ± 241.3 | 0.94 |
| **Fruits/KCal** | **Mean (±SD)** | 85.2 ± 55.9 | 169.0 ± 102.2 | 0.009 |
| **Vegetables/ KCal** | **Mean (±SD)** | 70.9 ± 40.6 | 93.4 ± 9.1 | 0.83 |
| **Glycemic load** | **Mean (±SD)** | 218.5 ± 90.8 | 278.0 ± 149.7 | 0.58 |
| **Glycemic index** | **Mean (±SD)** | 62.1 ± 7.1 | 65.3 ± 9.1 | 0.42 |
| **Sweetened beverages/ grams** | **Mean (±SD)** | 281.3 ± 273.0 | 262.1 ± 205.2 | 0.93 |
| **Fruit Juices Fresh /grams** | **Mean (±SD)** | 76.0 ± 99.3 | 24.2 ± 17.8 | 0.15 |
| **MEDICAL HISTORY** | | | | |
| **Hypertension diagnosis** | **Yes** | 1 (6.3) | 1 (9.1) | 1.00 |
| **Hypertension treatment** | **Yes** | 0 (0.0) | 1 (9.1) | 0.41 |
| **Dyslipidemia diagnosis** | **Yes** | 3 (18.8) | 3 (27.3) | 0.66 |
| **Dyslipidemia treatment** | **Yes** | 1 (6.3) | 2 (18.2) | 0.55 |
| **OBESITY INDICATORS AND VITAL SIGNS** | | | | |
| **Systolic blood pressure mmHg** | **Mean (±SD)** | 125.3 ± 12.5 | 123.4 ± 19.3 | 0.42 |
| **Diastolic blood pressure mmHg** | **Mean (±SD)** | 77.5 ± 7.6 | 77.9 ± 10.4 | 0.72 |
| **Heart rate (bpm)** | **Mean (±SD)** | 84.2 ± 10.2 | 77.4 ± 12.3 | 0.15 |
| **BMI (kg/m2)** | **Mean (±SD)** | 29.3 ± 4.9 | 33.4 ± 4.5 | 0.06 |
| **Waist circumference (cm)** | **Mean (±SD)** | 94.3 ± 10.5 | 109.4 ± 9.6 | 0.001 |
| **Body fat (kg)** | **Mean (±SD)** | 27.7 ± 11.2 | 36.7 ± 8.2 | 0.03 |
| **Muscle Mass (kg)** | **Mean (±SD)** | 27.5 ± 7.4 | 30.4 ± 8.3 | 0.37 |
| **LABORATORY MEASURES** |  |  |  |  |
| **HBA1C (%)** | **Mean (±SD)** | 5.5 ± 0.4 | 5.9 ± 0.2 | 0.03 |
| **Fasting plasma glucose (mg/dL)** | **Mean (±SD)** | 103.1 ± 7.2 | 105.5 ± 8.9 | 0.54 |
| **HOMA-IR** | **Mean (±SD)** | 6.4 ± 2.3 | 10.0 ± 2.6 | 0.003 |
| **Creatinine (mg/dL)** | **Mean (±SD)** | 0.7 ± 0.2 | 0.8 ± 0.1 | 0.22 |
| **Insulin (IU/mL)** | **Mean (±SD)** | 25.2 ± 9.1 | 37.8 ± 9.0 | 0.003 |
| **CRP (mg/dL)** | **Mean (±SD)** | 10.6 ± 6.2 | 16.2 ± 8.3 | 0.07 |
| **HDL-C (mg/dL)** | **Mean (±SD)** | 47.7 ± 16.4 | 40.9 ± 9.3 | 0.34 |
| **LDL-C (mg/dL)** | **Mean (±SD)** | 128.2 ± 45.0 | 106.1 ± 29.0 | 0.08 |
| **Triglyceride (mg/dL)** | **Mean (±SD)** | 149.8 ± 71.2 | 157.4 ± 40.7 | 0.58 |
| **Vitamin D (ng/dL)** | **Mean (±SD)** | 15.2 ± 8.6 | 16.4 ± 7.5 | 0.48 |
| **Urine microlbumin/creatinine ratio (ug/gm)** | **Mean (±SD)** | 412.0 ± 559.0 | 269.8 ± 505.1 | 0.70 |

LR: Low Risk of Diabetes or euglycemia; HR: High Risk for Diabetes or prediabetes; PD: Probable Diabetes.
